# B-cell and T-cell activation in South African HIV-1-positive non-Hodgkin’s lymphoma patients

**DOI:** 10.4102/sajhivmed.v19i1.809

**Published:** 2018-11-07

**Authors:** Brian T. Flepisi, Patrick Bouic, Gerhard Sissolak, Bernd Rosenkranz

**Affiliations:** 1 Department of Medical Biosciences, University of the Western Cape, South Africa; 2 Department of Medical Microbiology, Stellenbosch University, South Africa; 3 Department of Medicine, Division of Clinical Haematology, Stellenbosch University, South Africa; 4 Department of Medicine, Division of Clinical Pharmacology, Stellenbosch University, South Africa

## Abstract

**Background:**

Altered immune mechanisms play a critical role in the pathogenesis of non-Hodgkin’s lymphoma (NHL). HIV-1 (HIV) infection is associated with a state of excessive T-cell activation, which can lead to increased T-cell turnover and lymph node fibrosis.

**Objectives:**

This study aimed to determine the serum levels of circulating B-cell activation markers, and the expression of T-cell activation and regulatory markers in HIV-positive NHL patients.

**Method:**

The serum levels of circulating soluble(s) sCD20, sCD23, sCD27, sCD30 and sCD44 molecules, all of which are biomarkers of B-cell activation, were determined by enzyme-linked immunosorbent assays (ELISA), while biomarkers of T-cell activation (CD8+CD38+) and regulation (FoxP3) were determined by flow cytometry in 141 subjects who were divided into five groups: Combination antiretroviral therapy (ART)-naïve HIV-positive patients; ART-treated HIV-positive patients; HIV-negative NHL patients; HIV-positive NHL patients on ART; and healthy controls.

**Results:**

HIV-positive NHL patients had significantly higher serum levels of sCD20, sCD23, sCD30 and sCD44 than HIV-negative NHL patients, while all five biomarkers were significantly elevated in HIV-positive NHL patients when compared with ART-treated HIV-positive patients. HIV-positive NHL patients had higher CD8+CD38+ and lower FoxP3 expression than HIV-negative NHL and ART-treated HIV-positive patients.

**Conclusion:**

B-cell activation is increased in HIV-positive NHL patients and is associated with reduced regulatory T-cell populations and increased CD8+ T-cell activation.

## Introduction

Non-Hodgkin’s lymphoma (NHL) refers to a heterogeneous group of haematopoietic malignancies originating in the lymphocytes.^1,2,3^ Non-Hodgkin’s lymphoma is the second most common malignancy affecting HIV-1 (HIV)-infected individuals.^4^ Altered immune mechanisms play a critical role in the pathogenesis of NHL, as evidenced by increased rates of NHL among HIV-positive patients, transplant recipients and autoimmune disease patients.^5,6^ HIV infection has also been associated with a state of excessive T-cell activation, which has been shown to be a strong prognostic indicator for disease progression at different stages of HIV infection.^7^

Chronic immune activation during HIV infection leads to increased T-cell turnover and exhaustion and may precipitate lymph node fibrosis.^8^ The increased levels of both soluble biomarkers of inflammation and markers of T-cell activation have been shown to be associated with and predictive of increased morbidity and mortality in treated HIV infection.^9^ Previous studies indicated that those patients with the most marked B-cell activation are at increased risk of developing HIV-associated NHL.^10,11^ B-cell activation is characterised by lymphocyte proliferation, class switch recombination and somatic hypermutation, all of which are prone to DNA mutations that may lead to lymphomagenesis.^5^ There are two ways by which B-cell activation may occur: (1) interactions with activated T-cells, whose T-cell receptor recognises antigen presented by the B-cells or (2) activation by T-cell independent antigens.

In the current study, we examined serum levels of circulating B-cell activation markers and the expression of T-cell activation and regulation markers in HIV-positive NHL patients and in several comparison groups. Chronic immune activation has been suggested to be one of the mechanisms leading to the development of NHL in HIV-infected patients.^12^ Increased expression of CD38+ on CD8+ T-cells (CD8+CD38+) has been previously associated with immune activation, progression of HIV disease and death.^13^ In addition, CD8+CD38+ has been previously shown to function as a signalling molecule in B-cell chronic lymphocytic leukaemia (B-CLL) and has been linked with disease pathogenesis.^14,15^ CD8+CD38+ expression has been shown to be an important prognostic marker in B-CLL that is stable over time and is not significantly influenced by chemotherapy.^16^

## Materials and methods

### Study population

Patients diagnosed with HIV infection (ART-treated and naïve) and a CD4+ T-cell count of ≤ 350 cells/µL, patients with HIV-associated NHL, HIV-negative NHL patients and a healthy control group were included in this study. Study subjects were recruited from Tygerberg Hospital and Groote Schuur Hospital, both tertiary hospitals in Cape Town, South Africa, between October 2012 and February 2014. Blood samples were drawn. All subjects signed informed consent forms.

All subjects were aged 18 years and older. A total number of 141 subjects (61 males and 80 females) were recruited in the study. The study sample consisted of the following five age- and gender-matched groups: 16 healthy controls, 34 HIV-negative NHL patients, 31 HIV-positive NHL patients on combination antiretroviral therapy (ART), 28 ART-naïve HIV-positive patients and 32 ART-treated HIV-positive patients (Table 1). The mean age was 40 years. There were 53 Black, 61 mixed race and 27 Caucasian subjects. There were 48 active smokers, while 93 were non-smokers. The mean HIV viral load was 4905 copies/mL in HIV-positive NHL patients, 1044 copies/mL in ART-treated HIV-positive patients and 19 008 copies/mL in ART-naïve HIV-positive patients. The mean duration of ART treatment was 24 months in both ART-treated HIV-positive patients and HIV-positive NHL patients, while the mean duration of chemotherapy was three cycles in both NHL groups.

**TABLE 1:** Subject demographic characteristics.

Characteristics	HIV-positive NHL	NHL	HIV-positive ART	ART-naive HIV-positive	Controls
Total sample size	31	34	32	28	16
Age (mean)	40	50	40	35	34
**Gender**					
Male	13	14	13	14	7
Female	18	20	19	14	9
**Race**					
Black people	15	6	11	17	4
Mixed race	10	21	16	9	5
Caucasian	6	7	5	2	7
**Smoking status**					
Smokers	9	15	11	8	5
Non-smokers	22	19	21	20	11
HIV viral load (Mean)	4905	-	1044	19 008	-

HIV-positive NHL, HIV-positive non-Hodgkin lymphoma patients on combination antiretroviral therapy (ART); NHL, HIV-negative non-Hodgkin lymphoma patients; HIV-positive ART, ART-treated HIV-positive patients; ART-naive HIV-positive, ART-naïve HIV-positive patients; controls, healthy control subjects. Dimensions: total sample size = total number; age = years; gender = total number; race = total number; smoking status = total number; HIV-1 viral load = copies/mL.

### Determination of the T-cell activation and regulation markers

#### CD8+CD38+ expression

The expression of CD38+ on CD8+ T-cells (CD8+CD38+) was determined by flow cytometry as follows: 20 µL each of monoclonal antibodies anti-CD3 PerCP, anti-CD8 FITC and anti-CD38 PE (The Scientific group and Becton Dickinson (BD) Pty Ltd, Johannesburg, South Africa) were added into labelled BD Falcon tubes and mixed with 50 µL of whole blood. The tubes were vortexed gently and the samples incubated in a dark cupboard for 15 min at room temperature. FACS lysing solution (450 µL) was added, and samples were again incubated for 15 min under the same conditions. All samples were analysed on a BD FACSCanto II flow cytometer instrument by FACS Canto DIVA software immediately following incubation.

#### FoxP3 expression

FoxP3 expression was determined by flow cytometry as follows: 20 µL of anti-CD45-FITC, anti-CD3-PerCP and anti-CD4-APC (The Scientific group and Becton Dickinson (BD) Pty Ltd, Johannesburg, South Africa) were added to BD Falcon tubes labelled for each sample and standards of lymphocyte subsets (lymphosures) low and normal.^17^ The sample and lymphosures (100 µL) were added to the tubes containing antibodies, vortexed and incubated in a dark cupboard for 20 min at room temperature (20 °C – 25 °C). Following the incubation, 900 µL of FACS lysing solution was added, and samples were vortexed and incubated for 15 min under the same conditions. Samples were then centrifuged for 5 min at 2000 rpm.

The supernatant was decanted and the resultant pellet was resuspended in the residual volume of FACS lyse by vortexing gently. Phosphate-buffered saline (PBS) (2 mL) was added to all samples which were centrifuged at 2000 rpm for 5 min. The supernatant was decanted and the resultant pellet was resuspended in the residual volume of PBS. The FoxP3 Buffer C (500 µL) was added to all samples, and samples were vortexed and incubated in a dark cupboard for 30 min at room temperature. Samples were then centrifuged, the supernatant decanted and the resultant pellet resuspended in the residual volume of buffer C. FoxP3 PE antibody (20 µL) was added, and samples were vortexed and incubated in a dark cupboard for 30 min at room temperature. PBS (2 mL) was added, and samples were centrifuged at 2000 rpm for 5 min. The supernatant was decanted and the resultant pellet was resuspended in the residual volume of PBS. Then 5% fixative (50 µL) was added to all samples which were analysed within 24 h.

### Determination of serum levels of circulating B-cell activation markers

The serum levels of the following B-cell activation markers were assessed by enzyme-linked immunosorbent assay (ELISA) kit: sCD20 and sCD27 (CUSABIO, Houston, USA); sCD23, sCD30 and sCD44 (Biocom Africa [Abcam], Pretoria, South Africa). All assays were carried out according to the manufacturer’s protocol. All samples from each case and matched (age and gender) controls were tested together. The following basic principles of ELISA were followed: (1) Coating/Capture: direct or indirect immobilisation of antigens to the surface of polystyrene microplate wells. (2) Plate-blocking: addition of irrelevant protein or other molecule to cover all unsaturated surface-binding sites of the microplate wells. (3) Probing/Detection: incubation with antigen-specific antibodies that affinity-bind to the antigens. (4) Signal measurement: detection of the signal generated via the direct or secondary tag on the specific antibody.

### Data and statistical analysis

All data were captured and analysed using Microsoft Excel and Graph pad prism version 5 (San Diego, USA). Statistical analysis of in-transformed data was performed using one-way analysis of variance (ANOVA) with Bartlett’s test for equal variances. Analysis of the primary endpoint was performed using a Kruskal-Wallis with Dunn’s *posthoc* test. The study populations were regarded as independent variables and the specific marker value was regarded as dependent variable. Relationships between two continuous variables were analysed by logistic regression analysis and the strength of the relationship measured with the Pearson correlation, or Spearman correlation. A *p*-value of < 0.05 represented statistical significance in hypothesis testing and 95% confidence intervals were used to describe the estimation of unknown parameters.

## Ethical consideration

This study was approved by the health research ethics committees of Stellenbosch University (N12/03/015) and University of Cape Town (076/2013). All participants completed and signed the informed consent forms, which were available in English, Afrikaans and Xhosa.

## Results

### CD8+CD38+ expression

**FIGURE 1:**
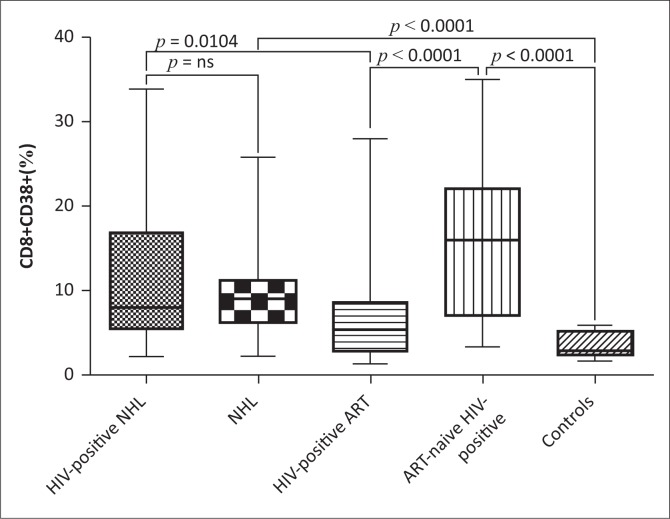
CD8+CD38+ expression (T-cell activation). HIV-positive NHL, HIV-positive non-Hodgkin lymphoma patients on combination antiretroviral therapy (ART); NHL, HIV-negative non-Hodgkin lymphoma patients; HIV-positive ART, ART-treated HIV-positive patients; ART-naive HIV-positive, ART-naïve HIV-positive patients; controls, healthy control subjects. Dimension: CD8+CD38+ = percentage (%).

CD8+CD38+ expression (%) was significantly upregulated in HIV-positive NHL patients on ART (HIV-1+NHL) as compared to ART-treated HIV-positive patients (HIV-1+ ART) (Mean ± s.d.: 10.8 ± 7.80 vs. 7.36 ± 6.90; *p* = 0.0104) (Figure 1). However, there was no significant difference between HIV-positive NHL patients on ART and HIV-negative NHL (NHL) patients. HIV-negative NHL patients had higher CD8+CD38+ expression than controls (Mean ± s.d.: 9.56 ± 5.53 vs. 3.65 ± 1.48; *p* < 0.0001). Antiretroviral therapy-treated HIV-positive patients had significantly lower CD8+CD38+ expression than ART-naïve HIV-positive patients (Mean ± s.d.: 7.36 ± 6.90 vs. 15.95 ± 8.81; *p* < 0.0001). Antiretroviral therapy-naïve HIV-positive patients had higher CD8+CD38+ expression than controls (Mean ± s.d.: 15.95 ± 8.81 vs. 3.65 ± 1.48; *p* < 0.0001).

### FoxP3 expression

**FIGURE 2:**
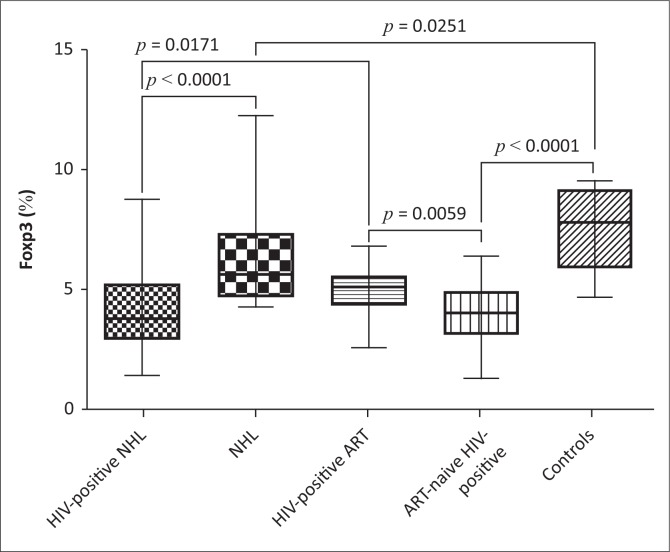
FoxP3 expression (T-cell regulation). HIV-positive NHL, HIV-positive non-Hodgkin lymphoma patients on combination antiretroviral therapy (ART); NHL, HIV-negative non-Hodgkin lymphoma patients; HIV-positive ART, ART-treated HIV-positive patients; ART-naive HIV-positive, ART-naïve HIV-positive patients; controls, healthy control subjects. Dimension: FoxP3 = percentage (%).

The expression of FoxP3 (%) was significantly downregulated in HIV-positive NHL patients on ART (HIV-positive NHL) as compared to both HIV-negative NHL (NHL) patients (Mean ± s.d.: 4.28 ± 1.87 vs. 6.37 ± 2.04; *p* < 0.0001) and ART-treated HIV-positive patients (HIV-positive ART) (Mean ± s.d.: 4.28 ± 1.87 vs. 5.02 ± 0.91; *p* = 0.0171) (Figure 2). HIV-negative NHL patients had significantly lower FoxP3 expression than controls (Mean ± s.d.: 6.37 ± 2.04 vs. 7.59 ± 1.70; *p* = 0.0251). As compared to ART-naïve HIV-positive patients, ART-treated HIV-positive patients had significantly higher FoxP3 expression (Mean ± s.d.: 5.02 ± 0.91 vs. 4.02 ± 1.28; *p* = 0.0059). In addition, ART-naïve HIV-positive patients had significantly lower FoxP3 expression than controls (Mean ± s.d.: 4.02 ± 1.28 vs. 7.59 ± 1.70; *p* < 0.0001).

### Serum levels of circulating B-cell activation markers

The serum levels (ng/mL) of circulating B-cell activation markers were significantly higher in HIV-positive NHL patients on ART (HIV-1+NHL) as compared to HIV-negative NHL (NHL) patients (sCD20 [Mean ± s.d.: 5.62 ± 1.69 vs. 3.92 ± 0.63; *p* < 0.0001], sCD23 [Mean ± s.d.: 3.39 ± 1.53 vs. 2.47 ± 1.56; *p* = 0.0192], sCD30 [Mean ± s.d.: 0.57± 0.25 vs. 0.38 ± 0.17; *p* = 0.0052], sCD44 [Mean ± s.d.: 7.25 ± 1.23 vs. 6.03 ± 1.41; *p* = 0.0014]) except sCD27, as well as when compared to ART-treated HIV-positive patients (HIV-positive ART) (sCD20 [Mean ± s.d.: 5.62 ± 1.69 vs. 4.75 ± 1.34; *p* = 0.0359], sCD23 [Mean ± s.d.: 3.39 ± 1.53 vs. 2.31 ± 2.17; *p* < 0.0001], sCD27 [Mean ± s.d.: 22.80 ± 11.20 vs. 13.71 ± 4.09; *p* = 0.0007], sCD30 [Mean ± s.d.: 0.57 ± 0.25 vs. 0.27 ± 0.26; *p* < 0.0001], sCD44 [Mean ± s.d.: 7.25 ± 1.23 vs. 4.84 ± 1.57; *p* < 0.0001]) (Table 2).

**TABLE 2:** Serum levels of B-cell activation markers (mean ± s.d.).

Biomarker	HIV-positive NHL	NHL	HIV-positive ART	ART-naive HIV-positive	Controls
sCD20	5.62 ± 1.69	3.92 ± 0.63	4.75 ± 1.34	5.11 ± 1.49	3.04 ± 0.84
sCD23	3.39 ± 1.53	2.47 ± 1.56	2.31 ± 2.17	1.15 ± 0.81	1.56 ± 0.59
sCD27	22.80 ± 11.20	22.28 ± 12.87	13.71 ± 4.09	19.74 ± 9.48	12.21 ± 1.87
sCD30	0.57 ± 0.25	0.38 ± 0.17	0.27 ± 0.26	0.26 ± 0.06	0.24 ± 0.12
sCD44	7.25 ± 1.23	6.03 ± 1.41	4.84 ± 1.57	6.08 ± 2.61	4.30 ± 1.37

HIV-positive NHL, HIV-positive non-Hodgkin lymphoma patients on combination antiretroviral therapy (ART)); NHL, HIV-negative non-Hodgkin lymphoma patients; HIV-positive ART, ART-treated HIV-positive patients; ART-naive HIV-positive, ART-naïve HIV-positive patients; controls, healthy control subjects. Dimension: Biomarker = ng/mL.

However, HIV-negative NHL patients had significantly higher serum levels of B-cell activation markers than controls (sCD20 [Mean ± s.d.: 3.92 ± 0.63 vs. 3.04 ± 0.84; *p* = 0.0025], sCD23 [Mean ± s.d.: 2.47 ± 1.56 vs. 1.56 ± 0.59; *p* = 0.0178], sCD27 [Mean ± s.d.: 22.28 ± 12.87 vs. 12.21 ± 1.87; *p* = 0.0033], sCD30 [Mean ± s.d.: 0.38 ± 0.17 vs. 0.24 ± 0.12; *p* = 0.0078], sCD44 [Mean ± s.d.: 6.03 ± 1.41 vs. 4.30 ± 1.37; *p* = 0.0013])] (Table 2). As compared to ART-naïve, HIV-positive patients, ART-treated HIV-positive patients had significantly higher serum levels of sCD23 (Mean ± s.d.: 2.31 ± 2.17 vs. 1.15 ± 0.81; *p* = 0.0074), lower serum levels of sCD27 (Mean ± s.d.: 13.71 ± 4.09 vs. 19.74 ± 9.48; *p* = 0.0038) and sCD44 (Mean ± s.d.: 4.84 ± 1.57 vs. 6.08 ± 2.61; *p* = 0.0130), while there was no significant difference in serum levels of sCD20 and sCD30. When compared to the controls, ART-naïve, HIV-positive patients had significantly higher serum levels of sCD20 (Mean ± s.d.: 5.11 ± 1.49 vs. 3.04 ± 0.84; *p* < 0.0001), sCD27 (Mean ± s.d.: 19.74 ± 9.48 vs. 12.21 ± 1.87; *p* = 0.0025) and sCD44 (Mean ± s.d.: 6.08 ± 2.61 vs. 4.30 ± 1.37; *p* = 0.0030), lower serum levels of sCD23 (Mean ± s.d.: 1.15 ± 0.81 vs. 1.56 ± 0.59; *p* = 0.0452), while there was no significant difference in sCD30.

### Associations between T-cell activation, regulation and B-cell activation

**FIGURE 3:**
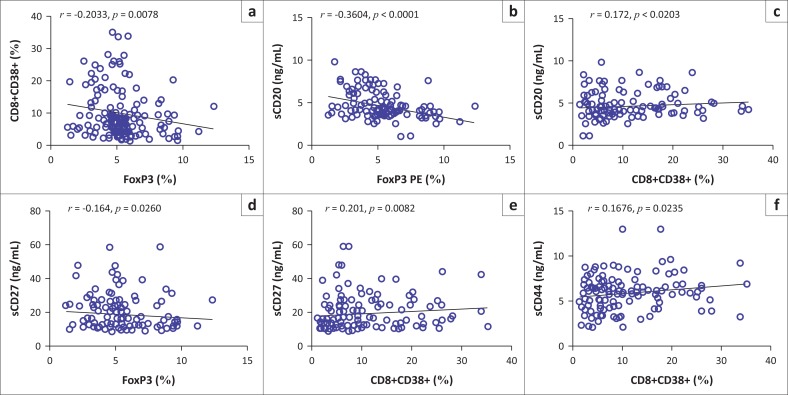
T-cell activation/regulation versus B-cell activation associations. (a) CD8+CD38+ (%) versus FoxP3 (%); (b) sCD20 (ng/mL) versus FoxP3 (%); (c) sCD20 (ng/mL) versus CD8+CD38+ (%); (d) sCD27 (ng/mL) versus FoxP3 (%); (e) sCD27 (ng/mL) versus CD8+CD38+ (%); (f) sCD44 (ng/mL) versus CD8+CD38+ (%).

The expression of CD8+CD38+ was negatively associated with FoxP3 expression (*r* = −0.2033, *p* = 0.0078) (Figure 3a). The serum levels of circulating sCD20 were negatively correlated with FoxP3 (*r* = −0.3604, *p* < 0.0001) expression (Figure 3b), while they correlated positively with CD8+CD38+ expression (*r* = 0.172, *p* = 0.0203) (Figure 3c). The serum levels of circulating sCD27 were negatively correlated with FoxP3 (*r* = −0.164, *p* = 0.0260) expression (Figure 3d), while they associated positively with CD8+CD38+ (*r* = 0.201, *p* = 0.0082) (Figure 3e) expression. The serum levels of circulating sCD44 were positively associated with CD8+CD38+ (*r* = 0.1676, *p* = 0.0235) expression (Figure 3f). No significant correlation was observed between sCD23, sCD30 and biomarkers of T-cell activation and regulation.

## Discussion

### Levels of CD8+ T-cell activation

In the current study, there was no significant difference in the expression of CD8+CD38+ between HIV-positive NHL patients on ART and HIV-negative NHL patients. However, although there was no significant difference, there was a trend towards increased CD8+CD38+ expression in HIV-positive NHL patients on ART. In addition, HIV-positive NHL patients on ART had significantly elevated T-cell activation as compared to ART-treated HIV-positive patients. Furthermore, as compared to the controls, HIV-negative NHL patients had increased CD8+CD38+ expression. The increased CD8+ T-cell activation observed in HIV-negative NHL patients may have been caused by Epstein-Barr virus (EBV) infection which has been associated with NHL or anti-tumour immune responses. CD8+ T-cell activation may be necessary for killing malignant lymphoma cells in NHL.^18^

To confirm if HIV infection leads to increased T-cell activation, the levels of T-cell activation between ART-naïve HIV-positive patients and control subjects were compared. T-cell activation was significantly elevated in ART-naïve, HIV-positive patients as compared to the controls and ART-treated HIV-positive patients. These results suggest that HIV increases T-cell activation and the ART initiation decreases T-cell activation. Deeks and colleagues^19^ reported that the initiation of ART during early HIV infection reduces the level of CD8+ T-cell activation. In addition, Almeida and colleagues^20^ showed that prior to ART, CD38+ expression was increased on peripheral blood CD8+ T-cells, and ART initiation significantly decreased CD38+ expression. Furthermore, the increased CD8+CD38+ expression was negatively associated with FoxP3 expression in the current study. This suggests that the increased T-cell activation observed in the current study may have been because of decreased T-cell regulation. The expression of FoxP3 which normally inhibits T-cell activation is reduced; thus, T-cell activation occurs continuously without regulation.

### Levels of T regulatory cells

FoxP3 plays an important role in regulatory T-cell (T-reg) function, development and maintenance.^21^ T-reg cells have been implicated in the suppression of T-cell activation, proliferation and cytokine production.^22,23^ Dysregulated T-reg cell expression has been associated with a number of pathological conditions including cancer, infectious and autoimmune diseases.^21^ In the current study, the expression of FoxP3 in HIV-positive NHL patients on ART was significantly downregulated as compared to HIV-negative NHL patients as well as when compared to ART-treated HIV-positive patients. The reduced FoxP3 expression observed in HIV-positive NHL patients on ART may have been caused by HIV infection, as the levels of FoxP3 expression were higher in HIV-negative NHL patients and ART-treated HIV-positive patients. This may have detrimental effects on T-cell regulation and activation.

FoxP3 expression was also downregulated in HIV-negative NHL patients as compared to the controls. This may have been caused by the direct effect of EBV on FoxP3 or the detrimental effect of immunosuppressive drugs such as cyclophosphamide, hydroxydaunomycin, oncovin and prednisone (CHOP) in the immune system. In a study conducted by El-Sayed and colleagues,^24^ it was shown that mRNA transcripts as well as percentages of FoxP3 were significantly increased in B-cell NHL patients before receiving CHOP, when compared to healthy controls. However, after six cycles of CHOP treatment, FoxP3 expression decreased significantly. These results suggest that T-cell regulation is impaired in both NHL and HIV-positive state.

As mentioned previously, one of the hallmark features of NHL is chronic immune activation. This may be because of suppressed T-cell regulation. In addition, the reduced T-reg cell expression observed in HIV-negative NHL patients may be beneficial as they may lead to increased immune activation and anti-tumoural responses, while the increased T-reg cell expression could limit the anti-tumour immune response, favouring tumour growth and development.^24,25^ To investigate the effect of ART on T-cell regulation, the levels of FoxP3 expression between ART-treated HIV-positive patients and ART-naïve HIV-positive patients were compared. The expression of FoxP3 was significantly increased in ART-treated HIV-positive patients than ART-naïve HIV-positive patients. Thus, ART may have increased the expression of FoxP3 in this population group. Consistent with the current findings, Andersson and colleagues^26^ reported suppressed FoxP3 expression in ART-naïve HIV-positive patients; however, upon initiation of ART, the levels of FoxP3 normalised. Antiretroviral therapy-naïve HIV-positive patients also had decreased FoxP3 expression as compared to the controls. This confirms that HIV infection decreases T-cell regulation, leading to chronic T-cell activation. T-reg cells have been shown to be susceptible to HIV infection.^27^

### Levels of B-cell activation

Soluble biomarkers of B-cell activation (sCD20, sCD23, sCD27, sCD30 and sCD44) were elevated in HIV-positive NHL patients on ART in the present study. Biomarkers of B-cell activation were also elevated in ART-naïve, HIV-positive patients, which indicates that chronic B-cell activation also occurred in untreated HIV-positive patients and ART may reduce B-cell activation. These results suggest that B-cell activation is increased in HIV-associated NHL as evidenced by increased B-cell activation markers investigated in this study. The increased B-cell activation has been previously observed in HIV-infected patients and was associated with a more rapid disease progression and poor survival.^8,10,14^ Breen and Colleagues^28^ reported that serum sCD23, sCD27 and sCD30 levels were significantly elevated in HIV-positive NHL patients as compared to HIV-positive controls.

Depleted FoxP3 expression was associated with increased T-cell activation. Increased B-cell activation was positively associated with increased T-cell activation and decreased T-cell regulation. The current findings indicate that chronic immune activation may have been a result of decreased immune regulation in HIV-positive NHL patients. Immune regulation is necessary in the control of immune activation and the prevention of auto immunity. T-reg cells are known to suppress T-cell activation, proliferation and cytokine production.^22,23^ In the absence of immune regulation, sustained immune activation occurs without monitoring. However, ART use was associated with improved immunity, increased regulatory T-cells and decreased T-cell activation.

The increased T-cell activation observed in HIV-positive NHL patients may have also caused chronic B-cell activation. It has been reported that chronic B-cell activation may be caused by related interaction with activated T-cells, whose receptor recognises antigen presented by the B-cells, or activation by T-cell-independent antigens.^29^ In addition, there is growing evidence that HIV virus can directly contribute to B-cell activation via direct interactions with B-cells.^14^ Chronic B-cell activation is known to increase the risk of HIV-associated NHL development.^5,10,30^ It has been previously shown that elevated serum levels of sC23, sCD27 and sCD30 are associated with subsequent diagnosis of HIV-associated NHL.^31^

The downstream effects of chronic B-cell activation with ongoing engagement of the B-cell receptor complex on lymphomagenesis are numerous and include the accumulation of oncogene mutations and translocations resulting from aberrant expression and gene targeting of the DNA mutating enzyme, activation-induced cytidine deaminase (AICDA).^31^ B-cell activation leads to the expression of AICDA, a DNA editing enzyme that mediates immunoglobulin gene class switch recombination and somatic hypermutation.^32^ It has been shown that AICDA is overexpressed before the development of HIV-associated NHL which is consistent with a direct role for this molecule in the pathogenesis of NHL.^32^

## Conclusion

Although CD4+ T-cell activation was not investigated, CD8+ T-cell activation is increased in NHL, as evidenced by increased CD8+CD38+ expression in HIV-positive NHL patients on ART as compared to ART-treated HIV-positive patients as well as in HIV-negative NHL patients as compared to controls. The influence of HIV infection on T-cell activation in HIV-positive NHL patients was not clearly defined in the current study, as there was no significant difference between HIV-positive NHL patients on ART and HIV-negative NHL patients. However, the current findings confirm that T-cell activation is greatly increased in untreated HIV infection. The observed chronic T-cell activation in HIV-infected patients may have been caused by a decreased regulatory T-cell expression as evidenced by decreased FoxP3 expression. This may lead to increased T-cell turnover and exhaustion, resulting in immune dysfunction. Antiretroviral therapy decreases T-cell activation while increasing its regulation.

B-cell activation is increased in HIV-positive NHL patients as evidenced by increased B-cell activation markers investigated in this study and is associated with reduced T-cell regulation and increased T-cell activation. The increased immune activation in this patient population group may have been caused by persistent HIV infection, as well as suppressed immune regulation. These findings confirm that B-cell activation is increased in untreated HIV-positive patients and in NHL patients. The serum levels of circulating B-cell activation markers are elevated in HIV-positive NHL patients and ART-naïve HIV-positive patients, and ART may decrease them. These data provide additional support for the recommendation that early ART initiation in all HIV-positive patients could be beneficial.

## Strengths and limitations

The sample size investigated in the current study was small; however, this was substantiated by formal statistical sample size calculation. The studied population groups were genetically diverse. HIV-positive patients had low CD4+ counts of ≤ 350 cells/µL, and CD4+ T-cell activation was not measured. HIV viral load was much lower in HIV-positive NHL patients and the mentioned EBV was not confirmed in NHL patients. B-cell activation was not measured directly; only soluble biomarkers of B-cell activation were measured. The current study provides important information on the levels of immune activation in South African HIV-positive, NHL patients.
